# A comparison of the wild food plant use knowledge of ethnic minorities in Naban River Watershed National Nature Reserve, Yunnan, SW China

**DOI:** 10.1186/1746-4269-8-17

**Published:** 2012-05-05

**Authors:** Abdolbaset Ghorbani, Gerhard Langenberger, Joachim Sauerborn

**Affiliations:** 1 Institute of Plant Production and Agroecology in the Tropics and Subtropics, University of Hohenheim, 70593, Stuttgart, Germany

**Keywords:** Edible plants, Biodiversity use, Cultural importance, Foraging

## Abstract

**Background:**

Wild food plants (WFPs) contribute to the nutrition, economy and even cultural identity of people in many parts of the world. Different factors determine the preference and use of WFPs such as abundance, availability, cultural preference, economic conditions, shortage periods or unsecure food production systems. Understanding these factors and knowing the patterns of selection, use and cultural significance and value of wild food plants for local communities is helpful in setting priorities for conservation and/or domestication of these plants. Thus in this study knowledge of wild food plant use among four groups namely Dai, Lahu, Hani and Mountain Han in Naban River Watershed National Nature Reserve ((NRWNNR), Xishuangbanna were documented and analyzed to find the similarity and difference among their plant use.

**Methods:**

Data on wild food plant use was collected through freelisting and semi-structured interviews and participatory field collection and direct observation. Botanical plant sample specimens were collected, prepared, dried and identified.

**Results:**

A total of 173 species and subspecies from 64 families and one species of lichen (*Ramalina* sp.) are used as WFP. There were differences on the saliency of wild food plant species among four ethnic groups. Consensus analysis revealed that knowledge of wild food plant use for each ethnic group differs from others with some variation in each group. Among informant attributes only age was related with the knowledge of wild food plant use, whereas no significant relationship was found between gender and age*gender and informants knowledge of wild food plant use.

**Conclusion:**

Wild food plants are still used extensively by local people in the NRWNNR, some of them on a daily base. This diversity of wild food plants provide important source of nutrients for the local communities which much of their caloric intake comes from one or few crops. The results also show the role of ethnicity on the preference and use of wild food plants. There is a big potential for harvesting, participatory domestication and marketing of WFPs especially in the tourism sector in the area.

## Background

Wild food plants (WFP) are plant resources that are harvested or collected from uncultivated resources for human consumption [1]. These plants are bestowed with one or more parts that can be used for nutrition if gathered at the proper growth stage and prepared appropriately [2]. WFP collection and use is still practiced in many parts of the world even among agricultural societies that rely mainly on domesticated plants and animals for their diet. In fact gathering wild plants is an internal part of livelihood strategies throughout the world [3]. WFPs are an important source of vegetables, fruits, tubers and nuts which are relevant for many people in ensuring food security and balancing the nutritional value of diets [1]. As an example, consumption of wild leafy vegetables as a source of micronutrients in many tropical areas is significant in small children’s diet to ensure normal growth and intellectual development [4]. However, different factors affect preference and use of WFPs such as abundance, availability, cultural preference, economic conditions, shortage periods or unsecure food production systems. Several WFPs are used only during food shortage or famine periods. Some are used on a daily base in one region or by a community while being considered as weed in other areas or by other communities. Understanding patterns of WFP use and cultural significance and value is important from cultural and nutritional perspective and also is helpful in setting priorities in conservation and/or domestication of these plants. It has also implications for rural development through marketing potential species and for people’s nutritional health by identifying nutritious species or promoting the use of wild food species. To achieve this, cultural domain studies are important. Cultural domain is a group of elements or items that is organized according to culturally determined rules or criteria and may be culturally specific, for example the domain of “medicinal plants” or “edible foods” [5]. Cultural domains are starting point for studying people’s perception of the natural world and are important aspects of local knowledge by which cultural organizations are understood [5]. Elements of a particular cultural domain (here WFPs domain) can be recorded and analyzed through free-listing interview methods [5,6].

The study area resides in Xishuangbanna Dai Autonomous Prefecture which is part of the Indo-Burma biodiversity hotspot, hosting 16% of China’s higher plant species, despite covering only 0.2% of the country’s land area [7,8]. The region is also culturally diverse with 13 different ethnic groups living within its territories. Because of this biocultural diversity many wild species are used by local population among them wild food plants. Xu et al. [9] reported 284 wild vegetables in Xishuangbanna comprising 6.1% of the total vascular plant flora. Chen et al. [10] also reported 123 species of wild edible fruits in Xishuangbanna.

Local people living in the Naban River Watershed National Nature Reserve (NRWNNR) benefit from a large number of forest products in their daily life. More than 182 species of food plants have been reported in NRWNNR [11]. The main wild food plant resources can be divided into vegetables, mushrooms and bamboo shoot categories. Zhang et al. [11] reported collection of bamboo shoots and mushrooms for income generation but vegetables were collected mainly for self-consumption. However information on the cultural importance of WFP species, patterns of WFPs use and knowledge variation among different ethnic groups living in the NRWNNR area is not available. This study aims to inventory and document WFP use knowledge in the NRWNNR and to compare WFP knowledge and use among Dai, Lahu, Hani and Mountain Han ethnic groups in the area and measure their cultural importance using some importance indices.

## Materials and methods

### Study area

**Figure 1:**
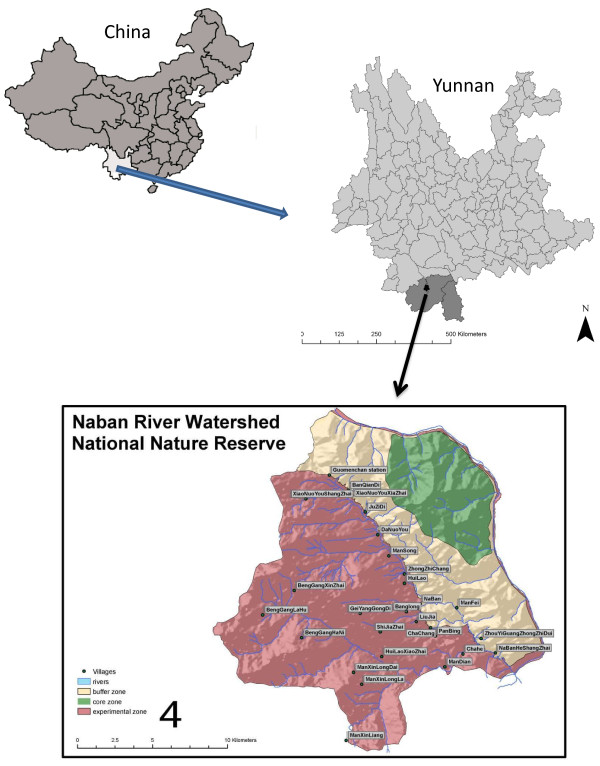
Location of study area and villages.

With a total area of 266.6 km^2^, NRWNNR is located in the central- north Xishuangbanna and lies on the west bank of Lancang (Mekong) River, approximately 25 km from Jinghong Township (Figure 1). It was established in 1991 based on the UNESCO’s “Man and Biosphere” concept. The nature reserve is divided into three functional zones; the core zone which is strictly protected from extractive activities, the buffer zone and the experimental zone which are both used for agricultural activities. However, any land use change in the buffer zone needs to be permitted by the nature reserve management office, which is not so for the experimental zone. NRWNNR harbors a plethora of biological as well as cultural diversity. More than 2345 species and subspecies of higher plants, 156 species of non-vascular plants, 437 species of vertebrates and 327 species of invertebrates are reported from NRWNNR area [12]. Six different ethnic groups including the Dai, Hani, Lahu, Yi, Bulang, and Mountain Han with a total population of 5538 people are living throughout the area [13]. In NRWNNR the Dai are living in three villages (Mandian, Naban and Manlei) which are located in valley bottom and lower elevations. Dai language belongs to the Tai (Zhuang-Dong) language family. The Dai have retained a very strong sense of ethnic cultural identity and are one of the ethnic groups best known to the Chinese people [14]. The Dai in Xishuangbanna have writing scripts that closely resembles the Thai script and they adhere to the Theravada Buddhist tradition. The Lahu of Yunnan Province began changing from hunter-gatherer lifestyle to settled village life in 1957 [15]. Lahu and Hani minorities are called hill tribes as their villages are normally located on higher altitudes than other minorities living in the same region. Lahu and Hani languages belong to the Tibeto-Burman language family and both are oral [14], however recently some efforts have been done to use Latin scripts for Hani language but Lahu language still has no scripts. Mountain Han group is also living in three villages of middle to high elevation and their language is a local dialect of Han Chinese language. Staple food in the area is rice (*Oryza sativa*) which is used along with different local vegetables and meat as protein source. Traditional lifestyle is still common in the area and people get many benefits from forest products.

### Data collection

Prior to starting the field work, research and plant collection permits have been applied for and issued by governmental officials and NRWNNR administration bureau. The research group was introduced to the village leaders and elders by NRWNNR administration officials and the objectives of the project were explained to obtain consent from them. Field survey was started in January 2008 and lasted for twelve months. Ethnobotanical data was collected through different interview methods [16-18]. Freelisting interviews with randomly selected informants were conducted. Freelists give information on salience, perception, classification and ranking of objects within a cultural domain in question, here WFPs [5,19]. In General, 217 Lahu, 129 Hani, 90 Dai and 49 Mountain Han individuals were interviewed. Semi-structured interviews, participant field collection and direct observation were followed to record data on the details of WFP local names, uses, collection, preparation and trade. Plant sample specimens were collected, prepared, dried, and identified with the help of experts from Xishuangbanna Tropical Botanical Garden (XTBG). One set of voucher samples was stored at the Herbarium of NRWNNR and one set was deposited at the Herbarium of XTBG (HITBC). Nomenclature follows Flora of China, Checklist of Flora of China, TROPICOS database of the Missouri Botanical Garden, and local checklists [20,21].

### Data analysis

Use frequency for each species was assessed by calculating citation frequency of species with respect to total number of interviews. Freelists were analyzed at the whole area level and at ethnic group level; according to frequency, average rank, salience and consensus using Anthropac 4.8. Anthropac calculates the frequency with which each plant is listed and its average rank in the freelists of each respondent then combines these to produce a measure of cultural importance or salience (Smith’s salience index) for each plant [22]. Species cited by at least two informants were considered for further analysis [22]. Freelist data then was dichotomized and a table of similarities with positive matches for plant species was constructed. Consensus analysis was conducted to analyze cultural variations among informants. Anthropac consensus analysis produces a hypothetical model of what correct answer to the freelist question would be or the shared knowledge of each group about WFPs. Then, the knowledge of informants is compared with this model and the degree of agreement to this model is calculated. If the reliability of the model is significant and the variation among informants is not high, the model represents the typical answer of a member of that population. Anthropac gives a reliability value (pseudo-reliability) and the closer is the value to 1.0 the higher the consensus among informants. The analyze was conducted for each ethnic group separately and also generally for the whole area. The results were compared between different ethnic groups. The relation between informant’s attributes (age and gender) and WFP knowledge was analyzed by ANOVA and further with Scheffe post-hoc test among 6 age groups. Sørensen similarity index was calculated among the different pair groups by EstimateS 7.5 and the similarity matrix was applied to conduct unweighted pair-group method using arithmetic average (UPGMA) cluster analysis using PC-Ord software to cluster ethnic groups. Microsoft Office Excel and SPSS 16 were used for further statistic analysis.

## Results and discussion

### Wild food plant diversity and frequently utilized species

A total of 173 species and subspecies belonging to 64 families and one species of lichen (*Ramalina* sp.) were mentioned by all four ethnic groups as WFP. *Rosaceae* was the most represented family (9 species) followed by *Zingiberaceae* (8 species) and *Araceae*, *Solanaceae*, *Poaceae* (7 species each). Commonly known species by all ethnic groups numbered 38. About 75% of species were common to the flora of China, 13% were endemic species and 12% were exotic and weed species. Most of the used plants are herbs (38.8%) followed by trees (24.8%), shrubs (19.7%), lianas (7%) and vine and culms (9.5%).

**Figure 2:**
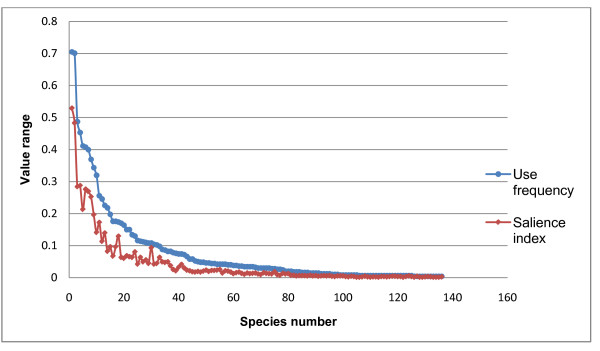
Comparison of consistency between use frequency and saliency of species.

Each informant mentioned 10.8 species in the list on average. More than 17% of the species were quoted only by one informant each. These low frequency species are considered either in passive use or used only in some idiolects [23]. The list of species cited by more than one informant is given in Additional file 1. Plant species which showed highest frequency of use include: *Diplazium esculentum* (Retz.) Sw. (use frequency = 0.7), *Musa accuminata* Colla (0.7), *Houttuynia cordata* Thunb. (0.48), *Ficus auriculata* Lour (0.40), *Oenanthe javanica* (Blume) DC. (0.45), *Solanum americanum* Miller (0.40), *Piper longum* L. (0.39), *Elatostema acuminatum* (Pair.) Brongn. (0.37), *Elsholtzia kachinensis* Parin (0.34) and *Bauhinia variegata* L. (0.31). The top ten frequently mentioned WFP and their salience among different ethnic groups are given in Table 1. These are also the most salience species. Frequently utilized species are generally corresponding with culturally important species (Figure 2). However there are also some slight variations. For example, value of Smith’s index for *Ficus auriculata* is less than *S. americanumP. longumE. acuminatum* (Table 1). This means that although *F. auriculata* is used more frequently than the other three species but these species are culturally more important than *F*. *auriculata* and ranked higher in the freelists. Most frequently utilized WFPs have also vast distribution range. Many WFP studies also show similar trend [24-27]. In fact these are common species which could be found easily around villages, crop fields and hedges. The Lahu WFPs include 95 species from which 18.9% are singly cited species and the average length of the list was 9 species. Hani use 123 species of WFPs from which 23.5% are cited by only one informant and the average length of list was 13.9 species. Dai instead use 95 species of WFPs, 20.2% of which cited by single informants and the average length of list was 10.5 species. The Han use 64 species, 28% mentioned only by one informant and the average list length was 10.9 species.

**Table 1:** Top ten frequently mentioned wild food plants and their salience at the whole area level and at ethnic group level

**Species**	**General**			**Lahu**			**Hani**			**Dai**			**Han**		
	**S**	**Use freq.**	**Rank**	**S**	**Use freq.**	**Rank**	**S**	**Use freq.**	**Rank**	**S**	**Usefreq.**	**Rank**	**S**	**Use freq.**	**Rank**
*Diplazium esculentum* (Retz.) Sw.	0.53	0.7	1	0.562	0.68	2	0.50	0.7	1	0.61	0.78	1	0.32	0.51	5
*Musa acuminata* Colla	0.48	0.7	2	0.561	0.76	1	0.35	0.56	4	0.54	0.77	2	0.47	0.73	4
*Oenanthe javanica* (Blume) DC.	0.287	0.45	3	0.25	0.39	6	0.42	0.59	2	0.30	0.54	4	0.30	0.51	6
*Houttuynia cordata* Thunb.	0.284	0.49	4	0.30	0.57	3	0.32	0.52	7	0.17	0.34	7	0.22	0.45	8
*Solanum americanum* Miller	0.27	0.41	5	0.24	0.3	7	0.38	0.54	3	0.27	0.46	5	0.14	0.18	11
*Piper longum* L.	0.26	0.4	6	0.21	0.35	5	0.10	0.18	21	0.46	0.58	3	0.32	0.49	5
*Elatostema acuminatum* (Pair.) Brongn.	0.25	0.37	7	0.30	0.46	4	0.23	0.34	9	0.05	0.11	22	0.53	0.65	2
*Ficus auriculata* Lour.	0.21	0.41	8	0.20	0.34	8	0.24	0.45	8	0.16	0.32	9	0.18	0.41	9
*Elsholtzia kachinensis* Parin	0.19	0.34	9	0.14	0.26	9	0.33	0.49	6	0.15	0.43	10	0.13	0.24	13
*Bauhinia variegata* L.	0.14	0.32	10	0.09	0.24	13	0.19	0.44	10	0.13	0.31	12	0.24	0.47	7

Species are ranked based on the Smith’s S index and ordered based on S index at the area level. *S* Smith’s S index, *Use freq.* use frequency.

### Species salience

There was variation on the saliency of species among groups when data was analyzed at group level. *Musa accuminata* (Smith’s S index = 0.56), *Diplazium esculentum* (0.52) and *Houttuynia cordata* (0.34) are top three salient species for Lahu (table 1). However among Hani, *D. esculentum* (0.51), *Oenanthe javanica* (0.49) and *Solanum americanum* (0.38) are the most salient species. Among Dai, *D. esculentum* (0.62), *M. accuminata* (0.54) and *Piper longum* (0.47) are the most important species and for Han *Schefflera brevipedicellata* Harms (0.54), *Elatostema acuminatum* (0.53) and *P. boehmeriifolium* (Miq.) Wall. ex C. Dc. (0.5) are the most salient species. These are also species which showed highest frequencies. The reason behind these variations might be the cultural preference of each ethnic group for special WFP. Pardo-de-Santayana et al. [25] compared the WFP knowledge in Iberian Peninsula and concluded that the patterns of WFP usage depends on socio-cultural factors rather than biological factors such as diversity of WFP, flora and climate. Chen et al. [10] also found that different ethnic groups in southern Yunnan consume wild fruits differently from each other. However, they conclude that environmental differences (and as a result difference in accessibility) and levels of agricultural productivity are reasons for different patterns of wild fruit use among ethnic groups. Geographical and environmental differences in southern Yunnan is coincident with ethnic group separation as Dai are living in valleys and at lower elevations consuming less wild fruits than other ethnic groups like Hani who are living in mountainous regions [10]. Termote et al. [28], by comparing the wild edible plant knowledge of three ethnic groups in Tshopo district of DRCongo, found that the use and knowledge of WFPs is culturally defined with high diversity between ethnic groups. In our study area, most of the salient species have vast distribution and are easily accessible. Nevertheless, it seems that differences in the ranking and saliency of species among ethnic groups are more related to the socio-cultural background than accessibility. It seems that use patterns of WFPs are strongly affected by culture. As an example, in the Amazonia or Eastern Europe wild green vegetables play a minor role whereas in East Asia and India, they are highly prized and large numbers of species are used [29].

### Edible plant part, growth form and use categories of WFPs

**Figure 3:**
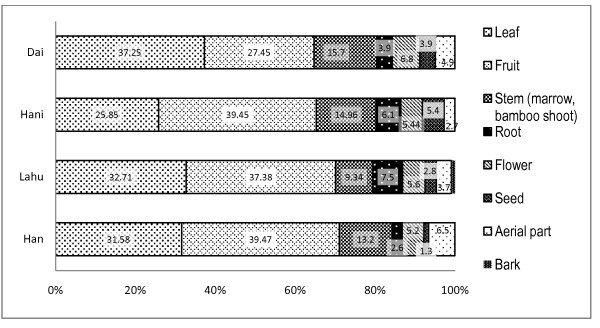
Comparison of plant part use of wild food plants among four ethnic groups (values represent percentage).

**Figure 4:**
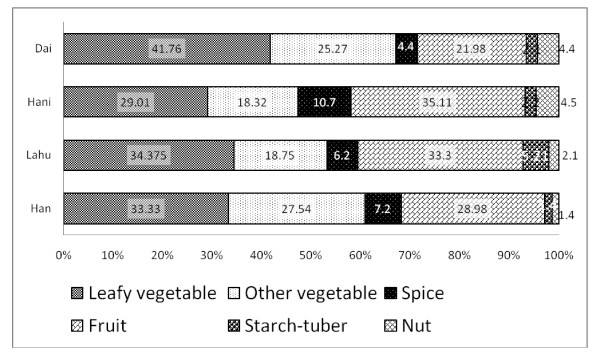
Comparison of wild food plant use categories among four ethnic groups (values represent percentage).

**Figure 5:**
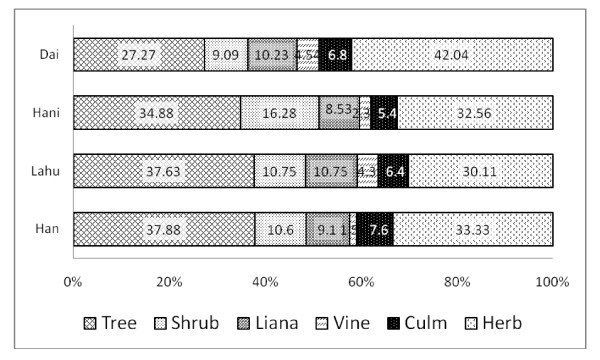
Comparison of plant growth forms used among four ethnic groups (values represent percentage).

Leaves of WFPs are the most common plant part (37.2%) mentioned to be used by Dai, however for the other three ethnicities fruits are the main plant part used (Figure 3). Fruit is the second commonly used plant part (27.4%) by Dai followed by stems (15.7%), flowers (6.8%) and aerial parts (4.9%). The other three ethnicities share almost similar pattern together as fruits being the most common used plant part followed by leaves and stems (Figure 3). WFP use categories also showed a similar pattern as Dai was different from other three groups. Leafy vegetables was the most common used category (41.7%) among Dai followed by other kind of vegetables (25.2%), fruits (21.9%) and spices (4.39%) (Figure 4). Lahu and Han WFP use showed a similar pattern. Among Hani the most common use category was fruits (35.1%) followed by leafy vegetables (29.01%), other vegetables (18.3%) and spices (10.6%). Selection of WFP’s life form among ethnic groups was in consistent with the use categories. That means the Dai who prefer leafy vegetables, tent to select herbs (42%) as WFPs while for the other ethnic groups, trees are the most commonly used life form (Figure 5). Cultural differences and habits as well as accessibility to the resources might be the reasons behind these differences. Because the Dai are living in lower elevations and most of their surrounding forests are almost converted to rubber plantations, they don’t have easy access to wild fruit trees in the forest. Some WFPs are known to be bound with cultural identity. For example, in the study area *Bauhinia variegata* L. is part of Dai culture and Dai people are known to eat flowers of this tree. *Rhus chinensis* L. is known to be part of Hani culture WFP.

### Similarity of WFP species between groups

**Figure 6:**
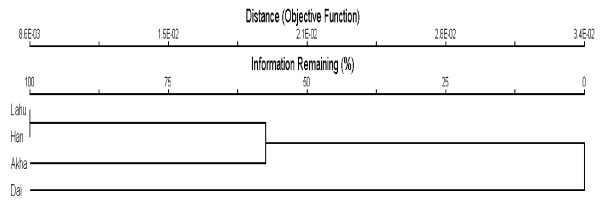
Dendrogram showing the result of clustering of four studied ethnic groups based on incidence of wild food species using UPGMA clustering.

The sørensen similarity index, calculated between ethnic groups based on incidence of common species revealed that Lahu and Han have highest values (0.713) which shared 56 species together. The Dai and Hani showed the lowest index value (0.544). Figure 6 shows the result of unweighted pair-group method using arithmetic average (UPGMA) cluster analysis based on sørensen similarity index. The dendrogram (Figure 6) indicates that Lahu and Han are grouped together and then Hani joins the cluster. This means that Lahu and Han share more WFPs maybe because they are living in the same village (XiaNoYou village) thus there should be an active knowledge exchange regarding WFPs. Then Hani joins the cluster. The Hani are also living in the higher elevations and having more or less the same access to the WFP resources. The Dai are living in lower elevations and in the rubber cultivation zone, so they might have different access to the WFPs than other groups.

### Informant consensus

Consensus analysis is a method of analyzing patterns of agreement among informants and finding the culturally correct answers to a set of questions. In Antropac, the knowledge of informants on WFPs is compared with a model and the degree of agreement is calculated. Table 2 shows the result of consensus analysis and also a list of key species used in the consensus model. The Lahu showed highest consensus (pseudo-reliability = 1) and higher mean estimated knowledge of 0.86 ± 0.06 and the Dai showed lowest mean estimate of knowledge (0.76 ± 0.09) indicating more diversity among the informants than the other three groups (Table 2). However all groups showed high pseudo-reliability (close to 1) meaning that informants have a higher consensus in the respective consensus key species. Factor loadings in eigenvalues table along with high pseudo-reliability imply that informants in each group are driven from a single culture [5]. Mengistu & Hager [30] also find similar results analyzing wild edible fruit knowledge of the Amhara region of Ethiopia. There was also overlap between salient species among each group and the ones included in the consensus model. In fact these are the species that are known to many people or used more often.

**Table 2:** Result of consensus analysis including freelist length, estimated informant knowledge, reliability value and species included in consensus key

**Ethnic group**	**Number of informants**	**Number of species mentioned**	**Mean freelist length**	**Mean estimate of informant knowledge**	**Pseudo- reliability**	**Number of species included in consensus model**	**Species fitting the consensus model**
Lahu	217	95	9	0.86 ± 0.06	1	3	*Diplazium esculentum* (Retz.) Sw., *Houttuynia cordata* Thunb., *Musa acuminata* Colla
Hani	129	123	13.9	0.82 ± 0.08	0.99	5	*Diplazium esculentum* (Retz.) Sw., *Houttuynia cordata* Thunb., *Musa acuminata* Colla, *Oenanthe javanica* (Blume) DC., *Solanum americanum* Miller
Dai	90	95	10.5	0.82 ± 0.09	0.99	5	*Diplazium esculentum* (Retz.) Sw., *Musa acuminata* Colla, *Oenanthe javanica* (Blume) DC., *Piper longum* L.
Han	49	64	10.9	0.76 ± 0.09	0.98	6	*Elatostema acuminatum* (Poir.) Brongn., *Musa acuminata* Colla, *Oenanthe javanica* (Blume) DC., *Piper boehmeriifolium* (Miq.) Wall. ex C. DC., *Piper longum* L., *Schefflera brevipedicellata* Harms

To find out the influence of informant attributes including age and gender on the knowledge of WFPs, an analysis of variance was conducted. Length of freelist was considered as knowledge proxy. The result illustrated that there was a significant relationship between age and the length of freelists, whereas no significant relationship was found between gender and age*gender (*p* > 0.05). Further multiple comparisons of age groups using scheffe post- hoc test revealed that knowledge of WFPs between age groups of 1 (10–20 years old) and 4 (41–50 y) and 5 (51–65 y) and also between 3 (31–40 y) and 4 (41–50 y) was significantly different (*p* <0.05). There was a clear difference on the mean of freelist lengths in each age group. Age group 4 and 5 had a similar mean list length of 12.2 species and age group 1 showed the lowest list length (9 spp). This could be interpreted as that younger people have less knowledge of WFPs and middle age people have more knowledge maybe because they are active in the collection and use of these species. It is a common believe that women have more knowledge of WFP than men because they are responsible for preparing household meals in many cultures, but our results show that in our study area there was no significant difference on the knowledge of WFPs between genders. Mengistu & Hager [30] also found age as the only attribute influencing wild fruit knowledge of the informants in Ethiopia. However, Watkins [31] documented that although knowledge scores of respondents were not significantly affected by gender and age among nomadic Turkana of northern Kenya, further analysis of male and female WFP lists showed interesting differences. These differences were related to WFP preparation methods. Male respondents mentioned WFPs which require little preparation while females mentioned WFPs that require special knowledge and more time to prepare.

### Trade of WFPs and sustainability

More than 45 species of WFPs are sold to the local markets occasionally (Additional file 1). However most of the WFPs are consumed in households and are not commercialized. Among the most dominant WFPs sold to the market are bamboo shoots of different species and *Eryngium foetidum* L., *Houttuynia cordataMusa accuminata* and *Bauhinia variegata*. Bamboo shoots are among the economically important and relative culturally important WFP group. Commercial exploitation of these plants without setting regulations on the collection practices may put pressure on the plant populations and at the same time cause conflicts between villagers. This is also true for *Houttuynia cordataMusa accuminata* and *Bauhinia variegata* which are all among most salience WFPs for all four ethnic groups. In case of bamboo shoots a regulation of sustainable collection has been set up by Nature Reserve Administration office, demanding a rotation period of two years. This regulation has not been implemented so far and villagers hardly follow these rules. Many villagers even complain about trespassers from other villages who exploit the resources from collective forests but there are no mechanisms to persecute these infringements. These highlights that for WFP which have cultural and economical importance among different ethnic groups a mechanism of management and harvest regulations should be implemented to prevent conflicts between villagers and also preserve the natural populations of these plants from overexploitation. Xu et al. [9] found 70 species of wild vegetables which are sold in the markets of Menglun, Xishuangbanna. These species of wild vegetables accounted for 30% of the total income from vegetable sales. They also found that most of the traders (95%) of wild vegetables in the market were women. Chen et al. [10] also recorded 17 species of wild fruits which are sold in local markets of southern Yunnan.

## Conclusions

Wild food plants are still used extensively by local people in the NRWNNR. This study provides an insight into the WFP knowledge and use patterns including culturally important and frequently used WFPs among four ethnic groups of the region. The area is rich in WFPs and our study also shows the dependency of WFP preference and use on culture, despite the WFP sharing among different ethnic groups. Although the studied groups are living in spatially different villages and this implies different accessibility to WFP resources, but the actual geographical distance between different villages is not so much that cause such a big geographical distance (Figure 1). On the other hand, species which are common among ethnic groups show different saliency ranking for each group (Table 1). This suggests the role of culture on the preference of WFPs.

Findings also show that highly salient species for the most part overlap with frequently used species. These are also the species most traded. There is a big potential for harvesting, participatory domestication and marketing of WFPs especially in the tourism sector in the area. Only in Jinghong City more than 100 restaurants cater wild vegetables for tourists [9]. Zhang et al. [11] also concluded that wild vegetable exploitation in NRWNNR could help for the economic development of the area. However this exploitation should be in a sustainable way and policies and regulations on exploitation of WFPs should be established. More investigation on the distribution patterns, population density and regeneration of these species could help planning and establishing harvest regulations that assure sustainable supply of plant materials.

## Competing interests

The authors declare that they have no competing interests.

## Authors’ contributions

**AG** conceptualized and designed the study, carried out field work and data collection and prepared manuscript. **GL** helped with the plant material identification and manuscript preparation. **JS** participated in study designing and manuscript preparation. All authors read and approved the final manuscript.

## Supplementary Material

Additional file 1 Wild food plants used by four ethnic groups in Naban River Watershed National Nature Reserve. Click here for file
