# Supplementary material for: A comparison of the wild food plant use knowledge of ethnic minorities in Naban River Watershed National Nature Reserve, Yunnan, SW China
Source: J Ethnobiol Ethnomed. 2012 May 5;8:17. doi: 10.1186/1746-4269-8-17 (PMC3485152; doi:10.1186/1746-4269-8-17)
Supplement: Additional file 1 — Wild food plants used by four ethnic groups in Naban River Watershed National Nature Reserve. [file 1746-4269-8-17-S1.doc]

Appendix1. Wild food plants used by four ethnic groups in Naban River Watershed National Nature Reserve.

| **Family** | **Scientific name** | **Local name** | **Growth form** | **Parts used** | **Use category** | **Preparation** | **Ethnicity**  **(used by)** | **Trade** |
| --- | --- | --- | --- | --- | --- | --- | --- | --- |
| *Acanthaceae* | *Thunbergia grandiflora* (Roxb. ex Rottel.) Roxb. | 藤子花 | Vine | L | Leafy veg | Fried / boil | L, Da | - |
| *Actinidiaceae* | *Actinidia umbelloides* C. F. Liang | - | Shrub | Fr | Fruit | Eaten raw | Hi | - |
| *Actinidiaceae* | *Saurauia funduana* Wall | 鼻涕果 | Tree | Fr | Fruit | Eaten raw | L, Hi, Ha | - |
| *Actinidiaceae* | *Saurauia* *napaulensis* DC. | 小鼻涕果树, 平地木 | Tree | Fr | Fruit | Eaten raw | L, Hi | - |
| *Actinidiaceae* | *Saurauia tristyla* DC. | - | Tree | Fr | Fruit | Eaten raw | Hi | - |
| *Amaranthaceae* | *Amaranthus spinosus* L. | 革命菜  (刺革命草) | Herb | L | Leafy veg | Fried / boil | Hi, Da | Y |
| *Amaranthaceae* | *Amaranthus viridis* L. | 玉米菜 | Herb | L | Leafy veg | Fried / boil | Hi, Da, Ha | - |
| *Anacardiaceae* | *Mangifera sylvatica* Roxb. | 林生芒果 | Tree | Fr | Fruit | Eaten raw | L, Hi, Da, Ha | Y |
| *Anacardiaceae* | *Rhus chinensis* Mill. | 盐酸果, 盐酸果树 | Shrub | Se | Spice | Added to the foods or chili sauce | Hi, Da | - |
| *Anacardiaceae* | *Spondias pinnata* (L.) Kurz. | - | Tree | Fr | Fruit | Eaten raw | L, Hi, Da, Ha | - |
| *Annonaceae* | *Annona squamosa* L. | - | Shrub | Fr | Fruit | Eaten raw | Da | - |
| *Annonaceae* | *Fissistigma polyanthum* (Hook. f. & Thomson) Merr. | 香木料藤 | Shrub | Fr | Fruit | Eaten raw | L, Ha | - |
| *Apiaceae* | *Centella asiatica* (L.) Urb. | 马蹄草 | Herb | Ap | Leafy veg | Boiled or eaten raw, used to make cold dish | L, Hi, Da, Ha | - |
| *Apiaceae* | *Eryngium foetidum* L. | 大芫细 | Herb | L | Leafy veg, Spice | Boiled or stir-fried | L, Hi | Y |
| *Apiaceae* | *Oenanthe javanica* (Blume) DC. | 水芹菜 | Herb | Ap, L | Leafy veg | Eaten raw with chili sauce, boiled or barbeque with fish | L, Hi, Da, Ha | Y |
| *Apocynaceae* | *Amalocalyx microlobus* Pierre | 酸扁果 | Liana | Fr | Fruit | Eaten raw | L, Hi, Da, Ha | Y |
| *Aquifoliaceae* | *Ilex umbellulata* (Wall.) Loes | 羊屎果树 | Shrub | Fr, L | fruit, leafy veg | Eaten raw with chili sauce or boiled | L, Hi, Ha | - |
| *Araceae* | *Alocosia* sp. | - | Herb | Tu | Starch-tuber | Boiled | L, Da | Y |
| *Araceae* | *Amorphophallus pachystylis* Hett | 山药 | Herb | Tu | Starch-tuber | Boiled | L, Hi | - |
| *Araceae* | *Arisema* sp. | 鸡爪菜 | Herb | L | Leafy veg | Fried | Da, Ha | - |
| *Araceae* | *Homalomena occulta* (Lour.) Schott | 野水芋 | Herb | St, L | Leafy veg | Cooked or roasted in bamboo | Da | - |
| *Araceae* | *Homalomena pendula* (Blume) Bakh.f. | - | Herb | St, L | Leafy veg | Cooked with *P*. *flaviflorum* or roasted in bamboo | Da | - |
| *Araceae* | *Lasia spinisa* (L.) Thw. | 大刺苞菜, 刺菜, 马桑官 | Herb | L, St | Leafy veg | Boiled or steamed | Hi, Da, Ha | - |
| *Araliaceae* | *Aralia armata* (Wall.) Seem. | 小刺苞菜, 大刺苞菜, 饿饭果 | Shrub | L | Leafy veg | Boiled | L, Da | Y |
| *Araliaceae* | *Eleutherococcus trifoliatus* (L.) S.Y. Hu | 无家菜, 无家菜 | Shrub | L | Leafy veg | Boiled or stir-fried, eaten with chili sauce | L, Hi, Da | Y |
| *Araliaceae* | *Macropanax undulatus* (Wall.) Seem | 七叶灵 | Tree | L | Leafy veg | Boiled | L, Hi, Ha | - |
| *Araliaceae* | *Schefflera brevipedicellata Harms* | - | Shrub | L | Leafy veg | Boiled and eaten with chili sauce, or pickled (about one week) | L, Hi, Da, Ha | - |
| *Araliaceae* | *Schefflera chinensis* (Dunn) H. L. Li | 树头菜, 龙爪树, 风湿药 | Tree | L | Leafy veg | Boiled /eaten raw | L, Ha | - |
| *Araliaceae* | *Trevesia palmata* (Roxb. ex Lindl.) Vis | 山月排, 马桑管果 | Shrub | Fl, Fr | Veg, Spice | Cooked or raw | L, Hi, Da, Ha | Y |
| *Arecaceae* | *Caryota monostachya* Becc. | 棕笋 | Tree | Fr | Fruit | Boiled / eaten raw | L, Hi, Da, Ha | - |
| *Arecaceae* | *Livistona saribus* (Lour.) Merr ex chev | 大白叶果 | Tree | Se, Fr, St | Nut, Fruit, Veg | Eaten raw / fried | L, Hi, Da, Ha | - |
| *Asteraceae* | *Crassocephalum crepidioides* (Benth.) S. Moore | 老胖草, 九柳光 | Herb | L, Ap | Leafy veg | Boiled or stir-fried and then eaten with chili sauce | L, Hi, Da, Ha | Y |
| *Asteraceae* | *Enydra fluctuans* Lour. | - | Herb | L, St | Leafy veg | Eaten raw | Da | - |
| *Asteraceae* | *Sonchus arvensis* L. | 苦马菜 | Herb | Ap | Leafy veg | Cooked | L, Ha | - |
| *Asteraceae* | *Bidens pilosa* L. | - | Herb | L | Leafy veg | Eaten raw | Ha | - |
| *Athyriaceae* | *Diplazium esculentum* (Retz.) Sw. | - | Herb | L | Leafy veg | Boiled or stir-fried, eaten with chili sauce | L, Hi, Da, Ha | Y |
| *Balsaminaceae* | *Impatiens aquatilis* Hook.f | 土各菜, 马鹿菜 | Herb | L | Leafy veg | Eaten raw with chili sauce | L, Hi, Ha | - |
| *Begoniaceae* | *Begonia dryadis* Irmsch. | 水葫芦 | Herb | L, St | Veg | Cook with fish | L, Hi, Da | - |
| *Bignoniaceae* | *Markhamia* *stipulata* (Wall.) Seem. | 毛尾木 | Tree | Fl | Veg | Boiled and stir-fried | L, Hi, Da | Y |
| *Bignoniaceae* | *Mayodendron igneum* (Kurz) Kurz | 马桑官果树, 芭拉花, 野杩木香藤 | Tree | Fl | Veg | Boiled and stir-fried | L, Hi, Da, Ha | Y |
| *Bignoniaceae* | *Oroxylum indicum* (L.) Kurz | 海船 | Tree | Fr | Veg | Boiled and stir-fried, eaten with chili sauce | L, Hi, Da, Ha | Y |
| *Boraginaceae* | *Trichodesma calycosum* Coll. ex Hemsl. | 鸡蛋花 | Herb | L | Leafy veg | Cooked or fried with egg and meat | L, Hi, Da | - |
| *Boraginaceae* | *Trigonotis peduncularis* (Trevis.) Benth. ex Baker & S. Moore | - | Herb | Fl, St | Veg | Fried | Da | - |
| *Burseraceae* | *Canarium tonkinense* Engl. | 青果 | Tree | Fr | Fruit | Smashed with chili /eaten raw | L, Hi, Da | - |
| *Capparaceae* | *Stixis* *suaveolens* (Roxb.) Pierre | - | Vine | Fr | fruit | Eaten raw | Hi | - |
| *Chenopodiaceae* | *Chenopodium ambrosoides* L. | 盐巴菜, 细地留 | Herb | L | Fruit | Eaten raw | L, Da | Y |
| *Clusiaceae* | *Garcinia cowa* Roxb. | - | Tree | Fr | Fruit | Eaten raw (sour taste) | Hi | - |
| *Cucurbitaceae* | *Cucumis hystrix* Chakr. | - | Herb | Fr, L | Leafy veg, Veg | Boiled or eaten raw | L, Hi, Da | Y |
| *Cucurbitaceae* | *Hodgsonia macrocarpa* (Blume) Cogn. | 野面瓜 | Liana | Se | Nut | Roasted and mixed with rice | L, Hi, Da | - |
| *Cucurbitaceae* | *Thladiantha grandisepala* A. M. Lu et Z. Y. Zhang | 皮肤病 | Herb | L | Leafy veg | Boiled | Da | - |
| *Dioscoreaceae* | *Dioscorea alata* L. | 山药 | Liana | Tu | Starch-tuber | Roasted / boiled / steamed / and then fried | L, Hi, Da, Ha | Y |
| *Ebenaceae* | *Diospyros kaki* L.f. | 海船, 野柿花 | Tree | Fr | Fruit | Eaten raw | L, Hi, Ha | - |
| *Elaeagnaceae* | *Elaeagnus conferta* Roxb | - | Tree | Fr | Fruit | Eaten raw | Hi | - |
| *Elaeocarpaceae* | *Elaeocarpus* *austroyunnanensis* Hu | - | Tree | Se | Nut | Eaten raw | Hi | - |
| *Euphorbiaceae* | *Aporusa* *yunnanensis* (Pax & K. Hoffm.) F.P. Metcalf | - | Tree | Fr | Fruit | Eaten raw | Hi | - |
| *Euphorbiaceae* | *Baccaurea ramiflora* Lour. | 小红孩, 三丫果 | Tree | Fr | Fruit | Eaten raw | L, Hi, Da, Ha | - |
| *Euphorbiaceae* | *Manihot* *esculenta* Crantz | 木薯 | Shrub | Tu | Starch-tuber | Boiled | L, Hi | - |
| *Euphorbiaceae* | *Phyllanthus emblica* L. | 橄榄树 | Shrub | Fr, Ba | Fruit, Spice | Fruit pickled, bark mixed with pork meat | L, Hi, Da, Ha | Y |
| *Euphorbiaceae* | *Sauropus androgynus* (L.) Merr. | - | Shrub | L | Leafy veg | Eaten raw | L, Hi | - |
| *Fabaceae* | *Acacia concinna* (Willd.) DC. | 酸棘棘 | Tree | L | Leafy veg | Boiled with egg / fried with egg | Da | - |
| *Fabaceae* | *Acacia* *pennata* (L.) Willd. | 臭菜, 大竹 | Tree | L | Leafy veg | Boil with egg / fried with egg | L, Hi, Da | Y |
| *Fabaceae* | *Bauhinia tomentosa* L. | 白花 | Tree | Fl | Veg | Boiled or stir-fried | L, Hi, Ha | Y |
| *Fabaceae* | *Bauhinia variegata* L. | 白花 | Tree | Fl | Veg | Boiled or stir-fried | L, Hi, Da | Y |
| *Fabaceae* | *Entada phaseoloides* (L.) Merr. | - | liana | Se | Nut | Pickled | Hi | - |
| *Fagaceae* | *Castanopsis clarkei* King ex J. D. Hooker | - | Tree | Se | Nut | Eaten raw | Da | Y |
| *Fagaceae* | *Castanopsis mekongensis* A. Camus | 大竹溜 | Tree | Se | Nut | Eaten raw | L, Hi, Da, Ha | Y |
| *Flacourtiaceae* | *Flacourtia ramontchi* L’Héritier, | - | Tree | Fr | Fruit | Eaten raw | Hi | - |
| *Gnetaceae* | *Gnetum* *montanum* Markgr. | - | Liana | Fr, Se | Fruit, Nut | Boiled, eaten raw | Hi | - |
| *Lamiaceae* | *Elsholtzia kachinensis* Prain | 水香菜 | Herb | L | Leafy veg | Eaten raw with chili sauce or boiled, cooked with dog meat or beef | L, Hi, Da, Ha | Y |
| *Lamiaceae* | *Pogostemon glaber* Benth. | 牛膝盖藤, 牛膝盖草 | Herb | L | Spice | Add to the dishes | Hi | - |
| *Lauraceae* | *Litsea cubeba* (Lour.) Pers. | 木姜子 | Tree | Fr | Spice | Add to the dishes | Hi | - |
| *Lauraceae* | *Phoebe puwenensis* W. C. Cheng | - | Tree | Fr | Fruit | Eaten raw | Hi | - |
| *Liliaceae* | *Polygonatum cirrhifolium* (Wall.) Royle | - | Herb | Rh, L | Veg | Boiled then eaten with chili sauce | Hi | - |
| *Marattiaceae* | *Angiopteris caudatiformis* Hieron. | - | Herb | R | Veg | Boiled | L, Da | - |
| *Marattiaceae* | *Angiopteris helferiana* Presl | 马蹄根 | Herb | R | Veg | Boiled | L | - |
| *Melastomataceae* | *Melastoma malabathricum* L. | 炸炮肚子果, 洋户官, 打破碗花树 | Herb | L | Leafy veg | Boiled | L, Hi, Ha | - |
| *Meliaceae* | *Toona sinensis* (A. Juss.) Roem. | - | Tree | L | leafy veg | Smashed with chili / pickled | Ha | - |
| *Menispermaceae* | *Parabaena* *sagittata* Miers | 梨板菜, 梨板叶 | Herb | L | Leafy veg | Eaten raw or fried or make soup | L, Hi, Da, Ha | - |
| *Moraceae* | *Ficus* *auriculata* Lour. | 象蹄叶, 象蹄菜, 五眼果 | Tree | L, Fr | Leafy veg, veg | Eaten raw or boiled | L, Hi, Da, Ha | Y |
| *Moraceae* | *Ficus* *hirta* Vahl | 鸡束果, 小饿饭果树 | Tree | L, Fr | Fruit | Boiled or eaten raw | L, Hi, Ha | - |
| *Moraceae* | *Ficus oligodon* Miq. | - | Tree | L, Fr | Fruit | Boiled or stir-fried | L, Da | - |
| *Moraceae* | *Ficus semicordata* Buch.-Ham. ex Sm. | 鸡束果 | Tree | L, Fr | Fruit | Boiled or stir-fried | L, Hi, Ha | - |
| *Musaceae* | *Musa* *acuminata* Colla | 芭蕉花 | Herb | Fl, Fr, St | Veg | Boiled and fried | L, Hi, Da, Ha | Y |
| *Myricaceae* | *Myrica esculenta* Buch. - Ham. ex D. Don | 杨梅果树 | Tree | Fr | Fruit | Eaten raw | L, Hi, Da, Ha | Y |
| *Myrsinaceae* | *Ardisia crenata* Sims | - | Shrub | Fr | Fruit | Eaten raw | Hi, Da | - |
| *Myrsinaceae* | *Ardisia* *virens* Kurz | 小和尚果 | Shrub | Fr | Fruit | Eaten raw | L, Hi | - |
| *Myrsinaceae* | *Embelia* *ribes* Burm. f. | 泡筒果藤,  泡筒果 | Shrub | St, Fr | Veg | Eaten raw | L, Hi | - |
| *Myrsinaceae* | *Embelia scandens* (Lour.) Mez | 酸头果 | Shrub | St, Fr | Veg | Eaten raw | Hi | - |
| *Myrtaceae* | *Psidium* *guajava* L. | 马梨甘 | Tree | Fr | Fruit | Eaten raw | L, Hi, Ha | - |
| *Myrtaceae* | *Syzygium szemaoense* Merr. & L.M. Perry | 羊屎果树 | Tree | Fr | Fruit | Eaten raw | L, Hi, Da | - |
| *Myrtaceae* | *Syzygium tetragonum* (Wight) Wall. ex Walp. | 干天果 | Tree | Fr | Fruit | Eaten raw | L, Hi, Ha | - |
| *Passifloraceae* | *Passiflora edulis* Sims | 西番莲 | Vine | Fr | Fruit | Eaten raw | L | - |
| *Piperaceae* | *Piper boehmeriifolium* (Miq.) Wall. ex C. DC. | 小麻疙瘩,  大疙瘩 | Liana | L | Leafy veg | Eaten raw or boiled | L, Hi, Da, Ha | - |
| *Piperaceae* | *Piper* *flaviflorum* C. DC. | 小疙瘩, 辣藤, 小疙瘩 | Liana | L | Leafy veg | Eaten raw or boiled | L, Hi, Da | Y |
| *Piperaceae* | *Piper longum* L. | 辣藤, 绿子叶 | Liana | L | Leafy veg | Eaten raw, fried with beef or pork, barbecued with bamboo or make soup | L, Hi, Da, Ha | Y |
| *Piperaceae* | *Piper sarmentosum* Roxb. | 辣藤, 绿子叶 | Herb | L | Leafy veg | Eaten raw or fried with pork, make soup | L, Hi, Da, Ha | Y |
| *Piperaceae* | *Piper yunnanense* Y.Q. Tseng | - | Herb | L | Leafy veg | Eaten raw, roasted, boiled, eaten with chili sauce | L, Hi | - |
| *Poaceae* | *Dendrocalamus brandisii* (Munro) Kurz | 黄竹笋 | Culm | St | Veg | boiled, stir-fried of pickled, eaten with chili sauce | L, Hi, Da, Ha | Y |
| *Poaceae* | *Dendrocalamus hamiltonii* Nees & Arn. ex Munro | - | Culm | St | Veg | boiled, stir-fried of pickled, eaten with chili sauce | L, Hi, Da, Ha | Y |
| *Poaceae* | *Dendrocalamus* *membranaceus* Munro | 泡竹笋 | Culm | St | Veg | boiled, stir-fried of pickled, eaten with chili sauce | L, Hi, Da, Ha | Y |
| *Poaceae* | *Dendrocalamus* sp. | - | Culm | St | Veg | boiled, stir-fried of pickled, eaten with chili sauce | L, Hi | Y |
| *Poaceae* | *Gigantochloa nigrociliata* (Buse) Munro | 薄竹 | Culm | St | Veg | Boiled and pickled | Da | Y |
| *Poaceae* | *Indosasa sinica* C.D. Chu & C.S. Chao | 苦笋 | Culm | St | Veg | Boiled | L, Hi, Da, Ha | Y |
| *Poaceae* | *Pleioblastus* *amarus* (Keng) Keng f. | 刺竹笋 | Culm | St | Veg | Boiled then eaten with chili sauce pickled then fried | L, Hi, Da, Ha | Y |
| *Polygonaceae* | *Fagopyrum dibotrys* (D. Don) H. Hara | - | Herb | L | Leafy veg | boiled or stir-fried, fried with egg, made soup with tomato and pork | L, Da | - |
| *Polygonaceae* | *Polygonum caespitosum* Bl. | 老金丹 | Herb | L, St | Leafy veg | Eaten raw | Hi | - |
| *Polygonaceae* | *Polygonum capitatum* Buch. -Ham. Ex D. Don | 老金丹 | Herb | L | Leafy veg | Eaten raw | Hi | - |
| *Pontederiaceae* | *Eichhornia crassipes* (Mont.) Solms | 干天果 | Herb | L | Leafy veg | Eaten raw with chili sauce | Da | - |
| *Pontederiaceae* | *Monochoria vaginalis* (Burm. f.) C. Presl ex Kunth | - | Herb | St, L | Leafy veg | Boiled then fried | L,Da | - |
| *Pteridaceae* | *Pteris wallichiana* J. Agardh | - | Herb | L | Leafy veg | Boiled then fried and eaten with sauce | Hi | - |
| *Rhizophoraceae* | *Carallia brachiata* (Lour.) Merr. | 斑鸠屎果 | Tree |  | Fruit | Eaten raw | L, Hi, Ha | - |
| *Rosaceae* | *Cerasus cerasoides* (Buch.-Ham. ex D. Don) S.Y. Sokolov | 樱桃 | Tree | Fr | Fruit | Eaten raw | L, Hi, Ha | - |
| *Rosaceae* | *Docynia delavayi* (Franch.) C.K. Schneid. | 哆依果 | Tree | Fr | Fruit | Pickled and eaten with chili sauce | L, Hi, Da, Ha | Y |
| *Rosaceae* | *Pyrus pashia* Buch.-Ham. ex D. Don | - | Tree | Fr | Fruit | Eaten raw | L, Hi, Ha | - |
| *Rosaceae* | *Rubus ellipticus* var. *obcordatus* Focke | - | Shrub | Fr | Fruit | Eaten raw | L, Hi, Da, Ha | - |
| *Rosaceae* | *Rubus alceifolius* Poir. | - | Shrub | Fr | Fruit | Eaten raw | Da | - |
| *Rosaceae* | *Rubus pluribracteatus* L.T. Lu & Boufford | 麻桑果 | Shrub | Fr, St | Fruit | Eaten raw | L | - |
| *Rosaceae* | *Rubus rufus* Focke | - | Shrub | Fr, St | Fruit, Veg | Eaten raw | Hi, Ha | - |
| *Rubiaceae* | *Toddalia asiatica* (L.) Lam. | 下鱼钩  小狗叶果 | Tree | Fr | Fruit | Eaten raw | Hi | - |
| *Rutaceae* | *Zanthoxylum myriacanthum* Wall. et Hook. f. | - | Shrub | Se | Spice | Cooked with food | Hi | - |
| *Sapindaceae* | *Nephelium chryseum* Blume | 毛李子果 | Tree | Fr | Fruit | Eaten raw | L, Hi, Da | Y |
| *Saururaceae* | *Houttuynia cordata* Thunb. | 鱼腥草  (狗青菜) | Herb | Ap | Leafy veg | Eaten raw or boiled, cold food, eaten with chili sauce | L, Hi, Da, Ha | Y |
| *Schisandraceae* | *Kadsura coccinea* (Lem.) A.C. Sm. | - | Tree | Fr | Fruit | Eaten raw | Hi | - |
| *Smilacaceae* | *Smilax china* L. | 山堆堆  老虎刺苔 | Vine | L, Fr | Leafy veg, Veg | Boiled or stir-fried | L,Da | - |
| *Smilacaceae* | *Smilax perfoliata* Lour. | 老虎刺苔 | Vine | L | Leafy veg | Eaten raw with chili sauce | Hi, Ha | - |
| *Solanaceae* | *Cyphomandra betacea* (Cav.) Sendt. | - | Shrub | Fr | Fruit | Eaten raw | Hi | - |
| *Solanaceae* | *Solanum americanum* Miller | - | Herb | L, Ap | Leafy veg | Boiled or stir-fried and used to make soup | L, Hi, Da, Ha | Y |
| *Solanaceae* | *Solanum spirale* Roxb. | 小苦了了 | Herb | Fr | Spice | Fried and smashed then added to chili sauce or added to the soup | L, Da, Ha | - |
| *Solanaceae* | *Solanum torvum* Swartz | 小苦果,  刺果树, 苦子果 | Herb | Fr | Spice | Fried and smashed then added to chili sauce | L, Hi, Da, Ha | Y |
| *Solanaceae* | *Solanum violaceum* Ortega | 小苦果,  苦果 | Herb | Fr | Veg | Fried and smashed then added to chili sauce | L, Hi | - |
| *Urticaceae* | *Elatostema acuminatum* (Poir.) Brongn. | 马鹿耳朵菜, 野巧菜 | Herb | L | Leafy veg | Boiled, raw or fried then eaten with chili sauce | L, Hi, Da, Ha | - |
| *Urticaceae* | *Oreocnide rubescens* (Blume) Miq. | 水麻 | Shrub | St | Veg | Boiled and then eaten with chili sauce | Hi | - |
| *Urticaceae* | *Pilea cadierei* Gagnepain & Guillemin | - | Herb | L. Ap | Leafy veg | Boiled and then eaten with chili sauce | Da | - |
| *Urticaceae* | *Pilea villicaulis* Hand.-Mazz. | 马鹿菜 | Herb | L, Ap | Leafy veg | Eaten raw | L, Hi, Da, Ha | Y |
| *Vitaceae* | *Tetrastigma obovatum* (M.A. Lawson) Gagnep. | 扁担果 | Vine | Fr | Fruit | Eaten raw | L, Hi, Da, Ha | - |
| *Zingiberaceae* | *Alpinia kwangsiensis* T.L. Wu & S.J. Chen | - | Herb | Fr, marrow | Spice | Eaten raw or fried | Hi | - |
| *Zingiberaceae* | *Amomum sericeum* Roxb. | 姜苗果 | Herb | Se | Spice | Add to the foods | L, Hi, Da, Ha | Y |
| *Zingiberaceae* | *Zingiber neotruncatum* T.L. Wu, K. Larsen & Turland | 野姜 | Herb | Rh | Spice | Cooked | L, Hi | Y |
| *Zingiberaceae* | *Zingiber trumcatum* S.Q Tong | - | Herb | Rh | Spice | Smashed with chili and used to make sauce | Hi | Y |

Used parts: Ap= aerial parts, L= leaves, Fl= flower, Fr= fruit, Se= seed, St= stem, Rh=rhizome, R= root, Tu= tuber, Ba= bark. Use categories: Veg= vegetable, Leafy veg= leafy vegetable. Ethnicity: Da= dai, Ha= han, Hi= hani, L= lahu. Trade: Y= traded
